# A Man Who Did Not Sweat

**Published:** 1878-10

**Authors:** A. R. Kilpatrick

**Affiliations:** Navastota, Texas


### A MAN WHO DID NOT SWEAT.

A. R. KILPATRICK, M. D.

Every one understands the importance of perspiration in preserving the body in a healthy condition, and maintaining an equilibrium of temperature. It is also one great channel for the cleansing of the system by excreting impurities, detritus and debris, which, if retained, would cause fevers, boils, or other morbid disturbances.

The sweat exudes through minute tubes, the outlets from small glands situated in the skin, and in some parts of the human body there are about five hundred of these tubes in every square inch of skin, while in other parts there are a thousand to the square inch, and in others there are about twenty-seven hundred in the same space. These tubes pass entirely through the skin, and do not follow a straight, direct course, but twist and turn in their transit, thereby increasing their length. Their united lengths aggregate a measurement of about one hundred and fifty-three thousand inches, or about two miles and a half. There are more of these tubes on the palms and soles than in

9. Always against the woman, you see.

other parts of the body. The amount of fluid thrown off varies in different individuals, and according to the conditions of quiet or exercise, as well as the temperature of the body and atmosphere. During violent exercise it has been as much as fourteen fluid ounces in an hour. Under ordinary circumstances, the average amount of perspiration by man in the course of twenty-four hours, is nearly two pounds avoirdupois.

Several years ago, there lived at Bayou Sara, West Feliciana Parish, La., a young German named Jacob Tieman, who had lately come from the Fatherland, and was working very hard as a drayman for a merchant in that place. He was honest, sober and industrious, and was allowed to eat at the table of his employer with the family, although he could not speak English. Jacob was cleanly in his habits, and every day, before dinner, he went down to the Bayou (creek), and under cover of the bushes, washed himself and got ready for his appearance at table. Finally, one day, he saw an empty flat-boat, or "broad-horn," moored near by his customary bathing place. Every few days these boats would be floated to Bayou Sara (town), some loaded with corn, flour, meat, &c., some with live stock, and some with lime in bulk or in barrels, and if the owners sold out there, they also sold the boat to some one, and they were usually torn to pieces and the lumber used for making outhouses, fences, &c.

Unluckily for Jacob, his new-found boat had lime in it, the floor, or bottom, was covered with it, but the boat was full of water, which was very clear, and looked so cool and tempting, like a nice bath-house, that he decided at once to make use of it, instead of the turbid Mississippi water in the Bayou Sara. The lime had clarified the water by precipitating the impurities, and it was as clear as freestone, or mountain spring water, but the lime was there in abundance. Coming as he did in the heat of the day, and perspiring freely from his laborious exercise, the pores of his skin were all agape and ready to imbibe anything. Jacob undressed rapidly, and dropped his reeking body into the grateful laver. However, he soon observed the white stuff in the water, and in a very short time felt a burning sensation in his skin, which caused him to quit sooner than he intended, and hastily don his clothing. His flesh smarted so he waited in the shade, but ere many minutes passed he became insensible, and was seized with violent fever. His absence from dinner was soon noticed, as he had always been so regular and prompt, and his employer, knowing his habit of bathing in the Bayou, apprehended that he was drowned, or attacked by an aligator, or snake, so he despatched some one to see after him, when he was found delirious and helpless. It is needless to tell of his long, painful and dangerous illness, through which he was attended by Dr. McKelvy, and finally restored to a degree of health.

From that time, he never had his skin moistened by perspiration, but was compelled to bathe regularly at short intervals, day and night, the year round. He afterwards, by industry and economy, accumulated enough to live easy, and he had a convenient bath fitted up in his house, which he used. He dressed in such a way that his clothes were removed easily—he also wore only slippers without socks. I have seen him, when he lived at Fort Adams, sixty miles below Natchez, on the Mississippi river, take an umbrella and spread it over him, while his body was stretched out in a shallow place in the great river, and remain there for hours, reading newspapers, or sleeping. As he grew old he became fleshy, although he was a very moderate eater, and drank very little beer or wine.

In the year 1846, he was compelled to attend District Court, as a witness, in a very important suit at law in Woodville, Miss. He plead earnestly with the Sheriff to let him off, telling him the condition of his health, (which the officer knew very well); the weather was extremely hot, being the month of August, and he said if he was deprived many hours of his accustomed bath, he feared he would be made sick, or perhaps it might kill him. When the time came, he explained his condition to the lawyers, and begged them not to detain him long; but they were like Gallileo, they "cared not for those things," and kept Uncle Jake several hours in the hot court room, and turned him out a short time before sunset. He had previously found a little brook in the outskirts of the town, where there was a hole of water in a hidden place, and he had resorted there to bathe; so, as soon as he left the court, he hastily repaired to it and laid down in the water, and soon fell into a deep sleep, from which he awoke about half-past 9 o'clock at night. When he reached town, nearly every house was closed, and many families had retired to bed.

Such was his life for about fifty years. He married a German woman, and they raised several healthy children. The cuticle resembled a cicatrix, or skin recently recovered from scalding, or a healing blistered surface. His case may be classed among the "Curiosities of Medical Experience."

NAVASTOTA, Texas.

